# Supplementary material for: Determinants of profiles of competence development in mathematics and reading in upper secondary education in Germany
Source: PLoS One. 2021 Oct 1;16(10):e0258152. doi: 10.1371/journal.pone.0258152 (PMC8486091; doi:10.1371/journal.pone.0258152)
Supplement: S1 File — (DOCX) [file pone.0258152.s001.docx]

Registered Report

Profiles of competence development in upper secondary education
and their predictors

- Online Supplement –

An additional online repository of the syntax files can be found at:

https://osf.io/x67bh/?view_only=77d0d99f497f43c3a2eb466f9b072553

This supplement offers additional information on changes in chapters 1-3 between the Registered Report Protocol and the final Registered Report (table S1), the descriptive statistics of all manifest variables (table S2) and the model comparison of the additionally conducted latent class growth analysis (table S3).

Table S1 provides all the changes between the background and methods in the original Registered Report Protocol and this Registered Report. For the most part this chapter remains exactly the same besides language updates (fitting to the new format), replacement of information and minor corrections in the procedure, all outlined in Table S1. This table is added mainly to increase the transparency of the changes made.

Table S2 provides a descriptive summaries of all manifest variables used in the imputation both prior and after imputation. The coding of the variables is explained in the Registered Report Protocol.

Table S3 offers a short overview over models with 1-10 profiles estimated with latent class growth analysis. This table can be read parallel to table 4 in the registered report for the BIC and group size. The LMRT was not provided here as a calculation with each of the 30 imputed datasets would be necessary for this calculation. This kind of calculation in combination with up to 10 profiles would necessitate estimation times exceeding reasonable efforts for an estimation not included in the original Protocol.

**Table S1: Changes in chapters 1, 2 and 3 relative to the Registered Report Protocol**

|  | **Chapter/s** | **Type of change** | **description** |
| --- | --- | --- | --- |
| **1** | 1.1 - 2 | Language (tense) | Minor changes of tense regarding expectations |
| **2** | 3.1 - 3.4.5 | Language (tense and context) | Changing language from planned to conducted context |
| **3** | 3.1 | Changing information | Replacing expected numbers with actual numbers |
| **4** | 3.1, 3.5 | Moving information | Moving information on data availability from chapter 3.1 into a new chapter 3.5 |
| **5** | 3.3 | Change in coding | Minor change in coding for specialization of vocational education - two dichotomized variables instead of one variable with 3 possible values |
| **6** | 3.3 | Removing information | Removal of descriptive results from the additional variables, the variables are described in Table S2 |
| **7** | 3.4.1 | Adding information | Adding the constraints of the slope parameters |

**Table S2: Summary of the main variables pre- and post-imputation**

| **Metric variables** | | | | | | |
| --- | --- | --- | --- | --- | --- | --- |
|  | | **Pre-imputation** | | | **Post-imputation** | |
|  |  | **Mean** | **SD** | **Miss.** | **Mean** | **SD** |
| Reading competence | Grade 9 | -0.03 | 1.26 | 1,115 | -0.04 | 1.26 |
|  | Grade 12 | 0.62 | 1.00 | 9,258 | 0.23 | 1.02 |
|  | Grade 12 + 3 years | 0.57 | 0.95 | 8,199 | 0.36 | 0.98 |
| Mathematical competence | Grade 9 | 0.02 | 1.21 | 489 | 0.01 | 1.21 |
|  | Grade 12 | 1.07 | 1.12 | 9,328 | 0.69 | 1.12 |
|  | Grade 12 + 3 years | 1.21 | 1.20 | 8,165 | 0.95 | 1.21 |
| Specialization of interest | | Generated after imputation | | | 0.00 | 1.33 |
| Highest CASMIN of parents | | 14.15 | 2.37 | 6,531 | 13.88 | 2.21 |
| Highest ISEI of parents | | 52.98 | 20.34 | 6,732 | 51.11 | 18.83 |
| Interaction language of students | | 0.23 | 0.52 | 432 | 0.23 | 0.52 |
| Age of students at first testing | | 15.16 | 0.64 | 31 | 15.16 | 0.64 |
| Self-concept in German | | 2.93 | 0.62 | 584 | 2.93 | 0.62 |
| Self-concept in mathematics | | 2.51 | 0.92 | 595 | 2.51 | 0.92 |
| Reasoning ability of students | | 8.65 | 2.46 | 1,108 | 8.60 | 2.44 |
| **Nominal variables** | | | | | | |
|  |  | **Cases** | |  | **Cases** | |
|  | **Reference Category** | **0** | **1** | **Miss.** | **0** | **1** |
| Reading specialized vocational education | < 6 months | Generated after imputation | | | 14,057 | 955 |
| Mathematics specialized vocational education | < 6 months | Generated after imputation | | | 10,392 | 4,620 |
| Gender | Girls | 7,471 | 7,527 | 14 | 7,478 | 7,534 |
| Migration background | No migration background | 10,841 | 2,919 | 1,252 | 11,965 | 3,047 |
| Type of school  in Grade 9 | Schools not leading to university entrance degree | 9,560 | 5,452 | 0 | 9,560 | 5,452 |

*Note*. The specialized interest and specialized vocational education could only be recoded after imputation. They therefore have no distributions outlined for pre-imputation.

**Table S3. Model comparison of the LCGA**

| Groups | *BIC* | Group size (based on estimated probabilities) | | | | | | | | | |
| --- | --- | --- | --- | --- | --- | --- | --- | --- | --- | --- | --- |
|  |  | P 1 | P 2 | P 3 | P 4 | P 5 | P6 | P7 | P8 | P9 | P10 |
| 1 | **191,161** | **15,012** |  |  |  |  |  |  |  |  |  |
| 2 | 225,597 | **8,420** | **6,592** |  |  |  |  |  |  |  |  |
| 3 | 221,630 | **7,732** | **4,793** | **2,487** |  |  |  |  |  |  |  |
| 4 | 220,383 | **6,445** | **5,004** | **2,493** | **1,069** |  |  |  |  |  |  |
| 5 | 219,963 | 5,731 | 4,918 | 2,622 | 1,262 | 479 |  |  |  |  |  |
| 6 | 219,646 | 5,665 | 4,850 | 1,645 | 1,255 | 1,144 | 453 |  |  |  |  |
| 7 | 219,368 | 4,377 | 3,448 | 2,757 | 2,081 | 1,153 | 799 | 397 |  |  |  |
| 8 | 219,047 | 3,372 | 2,975 | 2,751 | 2,644 | 1,297 | 1,027 | 661 | 287 |  |  |
| 9 | 218,796 | 3,142 | 2,808 | 2,697 | 2,409 | 1,425 | 1,141 | 572 | 562 | 256 |  |
| 10 | 218,686 | 3,003 | 2,837 | 2,309 | 2,233 | 1,610 | 1,314 | 721 | 512 | 333 | 139 |

*Note.* Bold indicates best values for a criterion, underlining indicates values being below our set threshold for acceptable profile size (> 5%).
