# Supplementary material for: Determinants of profiles of competence development in mathematics and reading in upper secondary education in Germany
Source: PLoS One. 2021 Oct 1;16(10):e0258152. doi: 10.1371/journal.pone.0258152 (PMC8486091; doi:10.1371/journal.pone.0258152)
Supplement: Attachment — Submitted filename: response to reviewers.docx [file pone.0258152.s002.docx]

Dear Dr. Kovanovic, dear reviewers,

together with this rebuttal letter, you may find the revised version of our manuscript entitled “Determinants of profiles of competence development in mathematics and reading in German upper secondary education”. We thank the reviewers for their thoughtful and helpful comments. The specific changes made regarding the editor and reviewer comments are described in detail in the tables below.

Comments in response to the Editor

| **Comments of the reviewer** | **Comments and changes to the manuscript** |
| --- | --- |
| We note that you have provided funding information that is not currently declared in your Funding Statement. | The “funding information” declared here is not the funding information regarding this manuscript but part of a mandatory acknowledgement needed to publish any article using NEPS data.  (see: <https://www.neps-data.de/Data-Center/Data-and-Documentation/Start-Cohort-Grade-9/Data-and-Citation>) |
| We noted in your submission details that a portion of your manuscript may have been presented or published elsewhere. […] Please clarify whether this [conference proceeding or publication] was peer-reviewed and formally published. If this work was previously peer-reviewed and published, in the cover letter please provide the reason that this work does not constitute dual publication and should be included in the current manuscript. | This previous Registered Report Protocol was indeed peer-reviewed and formally published at PLOS ONE (<https://doi.org/10.1371/journal.pone.0245884>). Prior to submission of this Registered Report we discussed this topic with an editor at PLOS ONE (Arianna Casabonne) who told us:  “After speaking with an internal editor about this, I can confirm that there is no plagiarism concerns for Registered Reports. It is acceptable to include the same materials and methods in your Registered Report that was included in your Registered Report Protocol. You can reuse text from the Registered Report Protocol, including the background. The Registered Report is a publication in its own right, a research article, and therefore should contain details on the background and methods as a regular Research Article would, regardless of what was already included in the Registered Report Protocol.”  Due to the specific nature of the Registered Report Protocol and the Registered Report, this should not be considered dual publication from our point of view. We additionally pointed out all changes between the Registered Report Protocol and the Registered Report in the Online Supplement to allow for maximum transparency. |
| In your revised cover letter, please address the following prompts:  a) If there are ethical or legal restrictions on sharing a de-identified data set, please explain them in detail (e.g., data contain potentially sensitive information, data are owned by a third-party organization, etc.) and who has imposed them (e.g., an ethics committee). Please also provide contact information for a data access committee, ethics committee, or other institutional body to which data requests may be sent. | We cannot offer the dataset, as it is indeed owned by a third party. The data is however available for researchers after a brief application procedure. We included the information of ownership and data availability in more detail in chapter 3.5. |

Comments and changes in response to Reviewer #1

| **Comments of the reviewer** | **Comments and changes to the manuscript** |
| --- | --- |
| Can you explain better what is the German National 171 Educational Panel Study (NEPS)? | We followed this advice and added some additional information in chapter 3.1. |
| What kind of activities have students to answer? | We addressed this question by adding a short statement on the inclusion of competence tests and questionnaires (3.1) |

Comments and changes in response to Reviewer #2

| **Comments of the reviewer** | **Comments and changes to the manuscript** |
| --- | --- |
| This is an interesting paper on the profiles of competence development in upper secondary education. The authors expected to identify generalised reading and mathematical competence profiles and unique profiles where one develops faster than the other. As a result, the profiles could then be predicted by students' interests and vocational education.  In this study, the results show only one relevant profile. The findings draw on the significant limitations of the study due to the results and included the omission of hypothesis two and three. In the conclusion section, the authors suggested that the competence profiles are questionable due to limitations in the analysis given the context of the study. This is an important finding to see if conditions hold true in other contexts. The overall methodology used to conduct the study is robust and the reviewer has no concerns present on the validity of the results. | We thank the reviewer for this overall positive evaluation of the paper. |
| This reviewer wonders if the single profile identified can be explained more in the discussion? | We added some additional information on the LGM (or singular profile) in the discussion. |
| Furthermore, what can the authors say about the results contradicting or corroborating with existing literature? | Using the information mentioned above, we also included more references to previous research and indicated whether findings were contradicting or corroborating our results. |
| What kind of recommendations for future research in this area can the authors give from the findings in this paper, such as avoiding the pitfalls and any other approaches that are needed to be explored? | While we had previously tried to answer this question as part of “Limitation”, we expanded this section in the manuscript. We especially included some recommendations for studies trying to find profiles of competence development similar to this study. |
| It is advised that colloquial terms in the manuscript are minimized where possible, such as could, would, should, may, etc. For example, line 127-128 contains “should therefore also more likely show”. This is but one example that requires a change to shorter and more assertive statements. Doing this will enrich the quality of the paper substantially. | Thank you very much for this note, we reduced the instances of these colloquial terms wherever possible.  We were, however, not able to take these instances out everywhere, but we checked to use it accordingly to differentiate hypothetical and certain statements. |

Comments and changes in response to Reviewer #3

| **Comments of the reviewer** | **Comments and changes to the manuscript** |
| --- | --- |
| I have carefully reviewed this manuscript (ms.). I must congratulate the authors of this ms. for several reasons: This work is robustly justified taking into account the previous empirical evidence found in Germany. Therefore, the objectives and the established hypotheses are consistent with the recruited sample in Germany. Along these lines, this reviewer wishes to congratulate the authors of this ms. since this fact is not common. Thus, this reviewer, after reviewing more than 150 ms. per year (many included in Q1, Q2 of the WoS-JCR) do not find articles as well organized (culturally focused), structured, written and robustly justified as this ms. Sincerely, congratulations! Furthermore, the type of sampling, the size of the sample and the statistical analyzes conducted are fine and up-to-date. It's GREAT! In any case, I would like to make some suggestions for the improvement of this ms., which can be published in PLOS ONE, of course: | We thank the reviewer for these kind words. |
| TITLE: The title of this ms. it should end by using the expression ".... in a sample of Germany students". This helps to quickly focus the reader of this ms. | Indicating the relevant population in the title is a good adjustment to improve the immediate accessibility of the paper. We thus added the notion “in Germany” to the title. Additionally, we adjusted for easier understanding to read: *Determinants of profiles of competence development in mathematics and reading in upper secondary education in Germany* |
| ABSTRACT Indicate type of sampling conducted. Indicate the name of the administered instruments. Specify type of statistical analysis conducted in this study. | We revised the abstract and added short information on all of these comments. |
| INTRODUCTION. This section should include more information regarding the German educational system. Authors should bear in mind that PLOS ONE is an international journal and, therefore, authors should be more precise when describing what the German educational system is like and what it consists of. This will undoubtedly make it even easier to understand this ms. by PLOS ONE readers. | As the description of the German educational system did indeed end up too short in the original manuscript, we extended this part by including some additional information.  Due to the convoluted nature of the German vocational education system – a high number of different school/education types, with different systems in every German state -, it was not possible to include all details in our manuscript, yet we refer to additional literature in which it is described in detail. |
| DISCUSSION Authors should robustly accept or reject the hypotheses initially raised in the Introduction section. | We fully addressed this comment by using clearer language to indicate that hypothesis 1 was rejected and hypothesis 2 and 3 were therefore not tested since they were conditional on H1. |
| Furthermore, it is essential that the authors present the main practical implications of the results found in this ms. both for researchers and applied professionals (mainly, school psychologists and other applied professionals). | We addressed this point at the end of the discussion with two additional paragraphs. |

In addition to the above mentioned changes, we made some minor additional changes and clarifications to improve the quality of the manuscript.

We hope the paper is revised satisfactorily in view of the reviewers’ detailed and thoughtful comments. We really appreciate your and the reviewers’ helpful feedback and effort. With the hopes that the present version is now acceptable for publication in PLOS ONE we remain

Sincerely,
Micha-Josia Freund, Ilka Wolter, Kathrin Lockl, & Timo Gnambs
